# Case of Epilepsy

**Published:** 1870-04

**Authors:** 


EPILEPSY.—Dr. Octave Huard, of New Orleans, U. S., reports to the *Gazette des Hopitaux* (Paris), the following statistics of a case of Epilepsy in a patient aged ten years, treated successfully by Bromide of Potass, viz.:

Approximate number of attacks from the beginning of the disease up to November 1st, 1867, 2000. November, '67, 59. December, '67, 84. January, '68, 116. February, '68, 83. March, '68, 105. April, '68, 87. May, '68, 98. June, '68, 129. Total 2743. Treatment with Bromide of Potass was commenced July 1st, 1868, since which the number of seizures have been as follows: July, 1868, 65. August, '68, 88. September, '68, 13. October, '68, 7. November, '68, 7. December, '68, 31. Total 211. Thus during three years previous to the beginning of the treatment the number of attacks was *about* 3000.

During the eight months immediately proceeding, *positively* 763 attacks. During the six months immediately after the commencement of the treatment, 211 attacks.

The diminution of the number of the attacks commenced, only after two months, when the system was nearly saturated with the medicine, that the improvement was manifested in a remarkable manner.

The increase in the number of seizures during December over November was due to the suspension of the medication. No attack having occurred in three weeks. — *Ibid.*

THERAPEUTICAL Society of Paris. M. Gubler recognized in two patients, a painter since dead, and a tinner still under treatment, epileptiform phenomena positively due to saturnine intoxication only; for there was no evidence, especially in the second patient, to show that these individuals were alcoholized.

It is somewhat difficult to establish clearly the differential characteristics of poisoning by lead and by absinthe in consequence of the epileptiform attacks.

In the first patient, the coma persisted more than twenty-four hours; then under a dose of 6 grammes of Bromide of Potassium this patient was partially relieved, then sunk gradually.

The second patient experienced very violent attacks, and was gradually restored by the use of Bromide of Potassium.— *Gazette Medicale.*

